# Children of parents with alcohol problems performing normality: A qualitative interview study about unmet needs for professional support

**DOI:** 10.3402/qhw.v11.30673

**Published:** 2016-04-20

**Authors:** Anne Werner, Kirsti Malterud

**Affiliations:** 1 Health Services Research Unit, Akershus University Hospital, Lørenskog, Norway; 2 Research Unit for General Practice, Uni Research Health, Bergen, Norway; 3 Department of Global Public Health and Primary Care, University of Bergen, Bergen, Norway; 4 The Research Unit for General Practice and Section of General Practice, Department of Public Health, University of Copenhagen, Denmark

**Keywords:** Alcohol, family secrets, childhood, health care, social interaction, dramaturgical metaphor, interviews

## Abstract

**Background:**

Children of parents with alcohol problems are at risk for serious long-term health consequences. Knowledge is limited about how to recognize those in need of support and how to offer respectful services.

**Method:**

From nine interviews with adult children from families with alcohol problems, we explored childhood experiences, emphasizing issues concerning potentially unmet needs for professional support. Smart's perspective on family secrets and Goffman's dramaturgical metaphor on social order of the family focusing on the social drama and the dramaturgy enacted by the children supported our cross-case thematic analysis.

**Findings:**

The social interaction in the family was disrupted during childhood because of the parent's drinking problems. An everyday drama characterized by tension and threats, blame and manipulation was the backstage of their everyday life. Dealing with the drama, the children experienced limited parental support. Some children felt betrayed by the other parent who might trivialize the problems and excuse the drinking parent. Family activities and routines were disturbed, and uncertainty and insecurity was created. The children struggled to restore social order within the family and to act as normally as possible outside the family. It was a dilemma for the children to disclose the difficulties of the family.

**Conclusion:**

Altogether, the children worked hard to perform a normally functioning family, managing a situation characterized by unmet needs for professional support. Adequate support requires recognition of the children's efforts to perform a normally functioning family.

Alcohol problems represent a major contribution to global health problems (Orford, Velleman, Natera, Templeton, & Copello, 2013; Rehm et al., 2009; World Health Organization, 2009), and affected families experience a heavy burden of stress and challenges (Balsa, Homer, & French, 2009; Barnard & Bain, 2015; Burke, Schmied, & Montrose, 2006; Hill, Laybourn, & Brown, 1996). Parental alcohol problems often lead to adverse childhood experiences with long-term health consequences (Balsa et al., 2009; Burke et al., 2006; Cleaver, Unell, & Aldgate, 2011/1999; Dube et al., 2001; Felitti et al., 1998). Children whose parents have alcohol problems are at risk for similar problems and severe medical and psychological conditions (Broning et al., 2012; Brown et al., 2009; Dube et al., 2001; Felitti et al., 1998; Orford et al., 2013; Rossow, Felix, Keating, & McCambridge, 2015). Yet, families with alcohol problems are heterogeneous, and not all children will develop significant problems (Järvinen, 2015; Rangarajan, 2008; Velleman & Templeton, 2007).

Within health policy and services, focus regarding alcohol problems has shifted from the individual problem drinker to the affected family, and family-focused and social network-focused interventions are recommended (Copello & Orford, 2002; Copello, Templeton, & Velleman, 2006; Velleman, 2010). Nevertheless, such approaches have often ignored the needs of affected family members themselves, and appropriate service provision to this group is still limited (Copello & Orford, 2002).

The family focus in the addiction field has also fostered discourses blaming or pathologizing affected families (Orford et al., 2013; Taleff & Babcock, 1998). Inappropriate stereotypes of these families and their mutual roles have been bred (Orford et al., 2013). Families with alcohol problems have been described as invisible, hidden or forgotten (Backett-Milburn, Wilson, Bancroft, & Cunningham-Burley, 2008; Gorin, 2004; Kroll, 2004; Velleman & Templeton, 2007; Wilson, Cunningham-Burley, Bancroft, & Backett-Milburn, 2012), and they seem to have experienced lack of information and support (Cleaver et al., 2011/1999; Mackrill, Elklit, & Lindgaard, 2012; Orford et al., 2013).

Improved coordination of health care and social services may lead to earlier recognition and treatment of alcohol problems as well as prevention of adverse childhood experiences (Dube et al., 2001). Talking with individuals who had attended professional support as grown-ups due to their parents drinking during childhood, we noticed that in spite of obvious needs, none of them seemed to have received professional help during their upbringing. Instead, they described having been betrayed by professionals and other adults who never asked about their situation or responded to their needs.

Adequate support aimed to prevent adverse childhood experiences requires knowledge about the children's needs and what might possibly keep them from disclosing parental drinking problems. Research about what is at stake in everyday life is limited. The question remains how to recognize those in need of service support and how to offer services paying due respect to their problems and efforts to solve them.

## Preconceptions and theoretical frames of reference

We wanted to provide a better understanding of everyday life challenges in families affected by parents’ alcohol problems, focusing on children's role and responsibilities. Below, we present conceptual and theoretical positions underlying our interpretations of social interaction in the family and potentially unmet needs of professional support among these children.

### Beyond stereotypes and psychologizing of families with alcohol problems

The concept *dysfunctional family* refers to impoverished emotional interactions within the family, characterized by avoidance of confrontations or inability to resolve conflicts (Haaken, 1993). Within such families, *co-dependency* has been coined as a notion to describe a pattern where family members develop an excessive sensitivity to the needs of others, including concealment of substance dependency and parentification of children (Haaken, 1993; Taleff & Babcock, 1998). Despite the vast literature discussing co-dependency, there is limited consensus on the definition of the term. Its impact on the treatment field has been strong (Orford et al., 2013). The concept has contributed to a much needed attention to those living with problem drinkers, but has been criticized for mediating disapproval, stereotyping and blaming the affected family members, pathologizing the caregiving role and the relationship (Haaken, 1993; Orford et al., 2013). We endorse the arguments of Orford and co-workers who have highlighted the disempowered position of the family and call for research and conceptual development of models better suited to explain the impact of alcohol problems on affected family members (Orford et al., 2013).

Feminist studies and family sociologists have paid attention to the home and family difficulties and emphasized the downside of privacy (Smart, 2007; Wilson et al., 2012). The sociologist Carol Smart discusses *family secrets*, calling attention to the belief that we all have secrets, indicating a cultural tolerance for and understandings of the need to keep secrets (Smart, 2011). Families present a specific face to the world and this appearance will not be the full story. Smart argues that these stories constitute the family and sustain kinship relationship. She also emphasizes cultural notions reproducing ideals of or assumptions about family life (Smart, 2007). The sociology of childhood has drawn attention to children within the structure of society and family relationship, acting as social agents and co-constructioners of their social world (Brady, Lowe, & Olin Lauritzen, 2015; Wilson et al., 2012). There is an extensive literature on the outcome for families living with alcohol problems. Still, the voices of children from these families are to a limited degree heard, and the costs of their coping efforts may be high (Backett-Milburn et al., 2008; Gorin, 2004; Hill et al., 1996; Järvinen, 2015; Kroll, 2004; Wilson et al., 2012).

### Social life as a drama of self—the interactional order(liness) of everyday life

The sociologist Erving Goffman (1922–1982) offered a dramaturgical metaphor to understand the interactional order of trivial everyday life and its implications for self (Goffman, 1959/1990, 1971/2010). Much can be learned about normal social conduct by carefully considering its abnormal forms (Smith, 2006). In “Insanity of Place,” he explores interaction when the internal social order of the family is disturbed (Goffman, 1971/2010). Face-to-face interactions rest on responsibilities and obligations. Individuals engage in *remedial interchanges*, intended to transform the offensive into acceptable by means of accounts, apologies and requests (Goffman, 1971/2010; Lemert & Branaman, 1997). As the grammar of conduct breaks down, *situational improprieties* are carried out by individuals refusing to maintain their social place as parents or spouses. Refusing to support the internal order and engage in remedial work, the offender creates *organizational havoc*, while the family tries to cover up. Goffman's dramaturgical perspective reveals the structural forces that turn individuals into performers, even when that is neither their aim nor desire (Lemert & Branaman, 1997). *Drama* indicates how individuals present faces to impress one another, while *dramaturgy* refers to the performed aspects of the presentation of self (Goffman, 1959/1990; Smith, 2006). Each individual attempts to construct the impression they have on others, struggling to offer an idealized impression of self and protecting themselves and the others from disrupted interaction. According to Goffman, human beings are not only strategic actors occupied with managing the impression other forms of them. It is only possible to define oneself in accordance with statuses, roles and relationships consistent with the social order. Goffman also speaks about *frontstage—*the public context where the performance is presented outwards to an audience, and *backstage—*the private domain where actors withdraw and prepare for frontstage performance (Goffman, 1959/1990).

In this study, we explored the family drama in everyday life and the childhood implications of families with alcohol problems. Descriptions of experiences, events and actions in terms of drama, dramaturgy and disturbance of the social order in the family provided insight into the performances of the other family members when parents develop alcohol problems. We have regarded the participants’ efforts to manage childhood situational improprieties while trying to maintain social order backstage and frontstage as dramaturgical strategies. Such strategies may have the purpose of aiming towards an idealized version of the family, with a potential impact on public recognition of the situation, implying possibly unmet needs for professional support.

### Situating the study

Knowledge about processes contributing to concealment or disclosure of drinking problems is needed to enable recognition of families with alcohol problems and understand the needs of professional support for the children. We would draw attention to childhood in families with alcohol problems neither pathologizing children's efforts to deal with the situation, nor stereotyping the family members’ strategies to manage. The challenges of exposing drinking problems need to be further explored. As a sociologist and a medical doctor, sharing an interest for marginality, we would approach social interaction beyond medical and psychological concepts and stereotypes, maintaining a perspective of how drinking problems may be conducted by all involved family members. Individuals are, however, socially and culturally connected, related and embedded. Smart's and Goffman's perspectives offer understanding of secrecies beyond personality and disease, observing cultural norms of social interactions and family life (Goffman, 1959/1990, 1971/2010; Smart, 2007, 2011). We apply the concept *alcohol problems*, without referral to diagnoses or objective measures. We talk about *the problem-drinking parent* and *the other (i.e., the sober) parent*, recognizing that these terms refer only to a limited aspect of their person.

The aim of the study was to explore childhood experiences in families with problem-drinking parents, emphasizing the impact of the social drama and the dramaturgy enacted by the children concerning their potentially unmet needs for professional support.

## Method

### Design, participants and ethical issues

We carried out a qualitative study based on interviews with adults who grew up in families with problem-drinking parents. Our sample included nine individuals, three men and six women aged 25–54 years (average 37 years). They were recruited to interviews by professionals at two alcohol and substance abuse clinics in Oslo/Norway. Four of the participants attended treatment services for family members, offered by these clinics. Five had previously joined these services.

We applied a purposive sampling strategy covering participants representing different demographical background. Four had fathers with drinking problems, and five had mothers with drinking problems. One of the participants had struggled with substance abuse himself. Five grew up in intact families with both birth parents. Two of the participants experienced their parents being divorced while they were young teenagers and two while they were in preschool age. Two were only child, while seven had one or more siblings. All were Norwegian born and permanently or temporarily employed, except one with disability pension. Socioeconomic status is presented in Table I. Five participants were living alone, while four were married or cohabitating. Together, they exemplify a broad diversity of backgrounds.

**Table I:** Participants—demographic background data.

	*N*
Age	
18–19	0
20–29	4
30–39	2
40–49	1
50–59	2
60–69	0
70+	0
Gender	
Women	6
Men	3
Children	
No	5
Expecting a child	1
Yes	3
Education completed	
Prim/sec school	1
High school	2
Bachelor level	3
Master level	3

The study was conducted according to the principles of the Helsinki Declaration (World Medical Association, 2013). The Regional Committee for Medical and Health Research Ethics concluded that the study was not regulated by the Health Research Act (ref IRB 0000 1870), and the study was approved by the local privacy protection advisors (Akershus University Hospital HF) (ref. 14–005).

### Data collection and analysis

The first author conducted all interviews during 2014. Interviews took place in the participants’ homes, the researcher's office, at one of the clinics, or another place by choice of participants, duration 60–120 min (average 86 min). The interviews were open-ended, semi-structured (Kvale, 1996) and based on an interview guide. The interviews dealt with childhood experiences in everyday life, and the impact of their parent's alcohol problem on their everyday lives during childhood and adolescence, and included experiences of service needs and offers. For the present article, we focused on the first issue, regarding this as the social context for service needs and offers. In later articles, we shall deal with the participant's experiences with professional services during childhood.

All interviews were digitally recorded, transcribed and analysed with systematic text condensation, a cross-case method for thematic analysis (Malterud, 2012). Both authors were involved and negotiated the four steps of analysis: (1) reading all the material to obtain an overall impression of the family context during childhood and recognize preliminary themes; (2) agreeing upon code groups from the preliminary themes, identifying meaning units representing different aspects of the everyday impact of the parents’ alcohol problems and coding for these; (3) establishing subgroups exemplifying vital aspects of each code group, condensing the content of each of them and identifying illustrative quotations; and (4) synthesizing the contents of the condensates for each code group by presenting a reconceptualized description of each category. Analysis was supported by Goffman's micro-sociological approach to interactional situations in everyday life, and his dramaturgical metaphor (Goffman, 1959/1990, 1971/2010). Smart's perspectives regarding relational sensitivity and family secrets enabled us to explore what was at stake in the everyday life of these children and their families (Smart, 2007, 2011). We searched for meanings or actions and events providing access to the backstage scene of the family as the context of the social drama experienced and managed by the children, and the subsequent unmet needs for professional support.

### Findings

The participants described how the social interaction of the family was disrupted during childhood because of their parent's drinking problems. An everyday drama characterized by tension and threats, blame and manipulation was the backstage of their everyday life. Apologies were never presented. Dealing with the drama, the children experienced limited parental support and also limited support from professionals and other adults. Some children felt betrayed by the other parent who might trivialize the problems and excuse the drinking parent. Family activities and routines were disturbed, and uncertainty and insecurity was created. The children struggled to restore social order within the family and to act as normally as possible outside the family. It was a dilemma for the children to disclose the difficulties of the family. Altogether, the children worked hard to perform as a normally functioning family, managing a situation characterized by unmet needs for professional support. Below, these findings are further elaborated, illustrated by quotations assigned fictive names of participants.

### The social drama inside the family—tensions and blame

The participants grew up in a variety of family situations. All of them described families with conflicts. Several had clear memories of a very tense atmosphere and the impression that a parent had drunk a lot. The problem-drinking parent was often in a volatile mood, initiating arguments or falling out with the other family members. Some said that when their parents were drunk, they would often become aggressive and threatening. Several portrayed the problem-drinking parent as unstable or irresponsible, behaving offended for trivialities and being cross and critical towards the children's behaviour, appearance and achievements. Others described a parent who wanted to be in charge, saying evil things, and then—maybe after an affective explosion—dismissed any conversation for days or weeks. The other family members were always assigned blameworthy by the problem-drinking parents, irrespective of what had happened:

From not having taken out the garbage, to getting lousy marks at school - or just being a mean person who did not say hello when coming home, or that my mother was cruel since she did not serve him a meal on his request … He calls you lazy and selfish, making him the only person in service, while you are like hell and the devil's kid. (Ann)

The participants gave details about how the problem-drinking parent had terrorized or manipulated the other family members. One stated that all the problems filled so much that the parent's assets did not compensate for all that other. Some mentioned the problem-drinking parent's two personalities within the family. One described her mother as the kind and good, who could also be a tyrant. Several remarked a conspicuous contrast between their parent's appearances within versus outside the family. Being charming and popular in public, while privately, a problem-drinking father would appear as a distinctly different person:

My dad comes in two versions: One very social one – loves to work and chatting with everyone, but he has no private friends. He drinks alone and never sees a friend at home. He is very isolated. But everybody knows him. (…) He is very chummy, except in the private sphere. (Fanny)

### Dealing with the havoc as the member of a twisted team

The children would not always agree with their parents’ ways of understanding and managing the family situation. Participants said the parents with drinking problems would never admit, but always deny problems, attributing all the troubles to the family members. They would always find an excuse, making their responses defendable. Apologies were never presented, the next day everything was normal and both parents behaved as if nothing had happened. One said:

If mum was very drunk, and I said, ‘Now you are drunk. Would you please go to bed? I cannot deal with this now’, she would stand there, saying ‘Oh, you always say I am drinking. I am tired after work.’ She would always say that I try to be mean with her, that there is something wrong with me - not her. She easily got offended. (Catherin)

Several participants described the other parent as being controlled by the problem-drinking parent, while trying to protect their children. The other parent would trivialize the problems and excuse the drinking parent. Some participants commented that the other parent and they themselves seemed to have lost the perception that the situation was alarming. Others described how lies were established to explain away the drinking problem. In many situations, the parents’ drinking problems disturbed family activities, routines and celebrations and created uncertainty and insecurity in everyday life for the children. The participants spoke about how they at early age together with their other parent tried to manage these challenges. One of the participants illustrated how her parents tried to pull the family together after a drinking episode:

That Christmas, like always, mum suddenly had a terrible hangover and would not be able to celebrate. Dad called and suggested that my sister and I should talk with mum to make her pull herself together, which he was not able to do. Mum made herself a victim, pitied herself and said: ‘I am really suffering’. She vomited, had diarrhoea and it was beyond all measure. Daddy - demonstrating his denial of the problem - asked me to go up and give mum a hug and wish her a happy Christmas. She destroys the Christmas, and I am supposed to give her a hug? I denied, dad was cross and blamed me for being difficult. (Betsy)

She added that her father never reacted, even when the children found their drunken mother lying on the bathroom floor with blood all over from having cutting herself on glass. She thought he just wanted to sweep the problems under the carpet. The participants felt betrayed by the other parent who would let it happen or lacked the capacity to draw a line instead of standing up and providing help. Two said, however, that their other parents had intervened, and one of them said she knew her mother had talked with her father about his drinking problems, but nothing helped. Her mother had also talked with health care professionals, but without any recognizable outcome. Another participant said:

It is a deficit of parental care, isn't it - not only from one, but also from the two of them? However, I do not blame my father - if so, I would also have to blame my mother with the drinking problem. But they often say that the other partner has a responsibility. I think my father thought that we would be able to get along. And in the end, he was actually also the one who took her to the doctor and pushed her to work. (…) My father did everything he could to keep the family together. (…) I believe they were preoccupied with doing well. (Gwen)

### Struggling backstage to restore social order and well-being for everybody

All participants emphasized how they struggled to manage the family problems during childhood. Contributing to the atmosphere in the home by surveillance and conducting the reactions of family members was an important task for most participants at young age. One of them said that not only her father, but also she, as a child, made sure that everything at home was okay. She watched out to prevent any of the elder siblings to get into conflict with their mother, who would then begin drinking. She described how she handled the situation:

I was Home Secretary as well as Minister of Foreign Affairs and put everything in order for my mother to prevent this bad attitude. I looked after everything. (Gwen)

Several of the participants said that they became accustomed not to provoke the problem-drinking parent by doing mistakes or stepping on the parent's toes. Some described a strategy where they became the family's mediators, telling their parents to stop quarrelling when things went bad. Some of them remarked that such responsibility was definitely not a choice they had made, but they felt they were expected to act like adults, in a vice-like grip with no other options. One participant said that, his father threatened him to stay with him when his mother moved out, and afterwards the son remained with his father:

If you follow her now, you will be responsible for the dissolution of the whole family. (David)

### Loyally performing frontstage appearance as normal as possible

Gradually, the participants suspected that the parents had alcohol problems, but the problems seldom were a topic for discussion at home. One explained that he had to be loyal and lie, while another referred to the secret that nobody outside the family ever should know about. A feeling of being different turned up for some of them. Especially during adolescence, it seemed critical to appear as normal as possible outside the family. Three participants described a strategic choice, conducting the secret about their problem-drinking parent very carefully to be popular with their classmates. Another said he had not really concealed the problems:

I never allowed my friends to come to my home. I never talked to anybody about it. I do not understand why. But I remember that during many years I was wondering whether it was normal that dad was drinking as much as he did. I do not think I actively have concealed the situation, because I did not know whether it was abnormal. (Eric)

Some participants emphasized uncertainty about whether their parents actually had an alcohol problem or if it was just normal. One said she was still not sure and that she had been afraid of being told that it was just something she had made up. Another mentioned sitting by the phone, almost calling the child welfare authorities several times, but refrained because she did not know what to say and felt so lonely with the problems. Some commented that people must have known, although nobody intervened. All emphasized experiences of being betrayed by adults and professionals, especially school teachers, who never asked about their situation or intervened, although they must have noticed problems. However, several also described their parents as clever at staying sober, hiding their problems in the outside world. One participant said that his father would always be able to talk himself out of any kind of trouble, while another told that his father just broke off the friendship if anybody tried to intervene regarding his drinking problems.

Some commented that interventions might have accelerated the problems, “…only creating more drama than necessary,” one said, “… when you have become as old as 16 years.” A participant mentioned that although her mother died several years ago, she still did not talk about the mothers’ drinking problems in respect of her father. Another spoke about being captured in a dilemma within the family:

It is difficult because it is your family. You fear that it will all go to pieces - although it has already been broken. The situation is hopeless because it is difficult to see any solution to it. (Ann)

Some participants reflected upon the dilemmas of concealing or disclosing the difficulties at home, referring to potential reactions in the neighbourhood. They emphasized that they risked disturbing the image of their own family as well as ideas of how parents are supposed to be if disclosing the drinking problems. One, who portrayed her family as “a good-looking family in a suburban harmony,” thought about how “the image of a parent as a safe rock” would be interrupted if she revealed the drinking problem. Another mentioned the taboo of speaking about parents’ drinking problems. People did not stand out that much, she said, taking care not to let her own trouble disturb the harmony of her friend's family:

You learn how to put a lid on the emotions and to restrain oneself to prevent provocations at home. And you notice what you are not allowed to talk about outside the house because it is taboo - because when you find yourself by a Friday evening treat with a friend's family, you feel the grief and losses and all the painful feelings of what you miss, but cannot mention because then you will intrude on the harmony of other families. (Fanny)

## Discussion

Our analysis revealed a difficult and dead-locked situation for children in families with drinking problems, indicating potentially unmet needs for professional support. The participants described a family situation with tension and threats, manipulation and blame, and they experienced limited support. Disclosing the problems would disturb the image of the family life. The children struggled to relate to the situation, to restore social order within the family and to perform normality outside the family. Below, we discuss the strengths and limitations of the study design and the impact of these findings.

### Strengths and limitations

To recognize the needs among children from families with alcohol problems and improve medical and social services for this group, knowledge about affected children's experiences is required. We decided to talk with children at adult age, approaching their retrospective versions of the family histories, rather than discussing these issues with youths situated in the middle of the problems. This was a strategic choice, since a core aspect of the problems we wanted to study was drinking problems that might not be acknowledged and known to the services. A consequence of interviewing adults about childhood experiences is that stories are modified and worked through over time, shaped by the way these experiences are digested and elaborated (Good, 1994). For some of the participants, the actual events have taken place several years ago. We have taken their accounts as personalized, situated and elaborated versions of family life, emphasizing their interpretations of the family situation and parental alcohol problems as the context of our study. Acknowledging that experiences are transformed by time, we still assumed that adults would be able to reflect upon the implications in retrospective and to think about needs, services and offers. The majority of participants were women, well educated and childless at the time of the interviews. A broad range of demographic variables, family experiences and both mother and fathers with drinking problems and various drinking patterns were included in our sample. All participants had been recruited from treatment groups for members of families with alcohol problems. While the professional support during childhood had been modest within our sample, all of them later felt a need for help and contacted a specific service. Compared to other children from families with alcohol problems, our participants may hence have experienced a larger burden of repercussions. Most of them, however, appeared to be functioning well regarding personal life, education and work. Our participants may also be extraordinary since they have been able to seek support at adult age. Their participation in group treatment could have influenced their stories, for example, by making the experiences and strategies they presented appear more similar. Nevertheless, their stories presented a diversity of aspects regarding experiences and needs. It might further be objected that the other actors on the scene have not been requested their version. The ethical challenges of interviewing parents who did not view their alcohol habits as drinking problems would, however, be substantial.

Disclosing stories about personally taxing events and interactions, the participants will offer a presentation of self-corresponding with their current self-image, maybe moderating less flattering merits (Goffman, 1959/1990, 1971/2010). We found the participants’ stories to be quite detailed, some of them very considerate regarding the other family members, and often reflective concerning their own role in the family drama. The participants’ accounts gave us access to the study of family interactions from an insider perspective of marginality. Recruiting adult participants also gave the benefit of a time span of life experiences adding reflective perspectives on the consequences of the situation, especially regarding support from outside the family. Our point of departure was a parent's drinking behaviour which in retrospect was identified as a problem by the affected child, regardless of whether the parents would have agreed or whether physical symptoms of dependency were present. The very specific descriptions of drinking behaviour, subsequent problems and lack of support in the participants’ accounts, including experiences of having been betrayed, substantiate the study context as families with alcohol problems’ unmet needs for professional support.

Our analysis was supported by symbolic interactionism (Goffman, 1959/1990, 1971/2010) emphasizing the social interaction in the family as perceived by the children (Brady et al., 2015; Smart, 2007, 2011). This encouraged a gaze based on an understanding that the children's actions and strategies were intentional aiming towards restoration of the social order in some way or other (Goffman, 1971/2010), living up to ideals for or assumptions about a normal family life (Smart, 2007).

### What is known from before—what does this study add?

We are not the first to report experiences of stress and conflicts, tension and threats, coping dilemmas, concealment, uncertainty and worry among family members living with substance abuse (Backett-Milburn et al., 2008; Gorin, 2004; Haugland, 2005; Hill et al., 1996; Kroll, 2004; Orford et al., 2013; Rossow et al., 2015; Velleman & Templeton, 2007; Wilson et al., 2012). A review of research about experiences among children living with domestic violence or parental substance abuse concluded that the trouble at home was often kept as a family secret (Gorin, 2004). Children used different strategies to deal with such situations, such as avoidance/distraction, protection/inaction, confronting, intervention and self-destruction, or help-seeking and action. Our data confirm research demonstrating unmet needs of families in troubles, disruptions in everyday rituals and routines, and family members trying to find the best way of dealing with the situation (Backett-Milburn et al., 2008; Haugland, 2005; Mordoch & Hall, 2008; Orford et al., 2013; Trondsen, 2012; Trondsen & Tjora, 2014). Roles within these families are distorted, with the children responsible for paying attention and making one's arrangements in accordance with the parent with drinking problems, taking on caring and protective roles within the family (Haugland, 2005; Hill et al., 1996; Kroll, 2004; Vernig, 2011).

Families affected by drinking problems have been described as dysfunctional, comprising a standard set of roles, such as enabler, hero, lost child, mascot and scapegoat, with their strategies to manage explained as co-dependency (Haaken, 1993; Vernig, 2011). Our study adds to existing research by describing the complexity in the children's role and responsibility in managing dilemmas, challenges and strains in families with alcohol problems. These circumstances, as reported from the adult children's perspectives, indicate unmet needs for professional support and suggest why recognition and intervention are not straightforward measures to carry into effect.

Furthermore, our analysis have demonstrated how Smart's perspectives on *family secrets* and Goffman's dramaturgical metaphor and perspectives on *the havoc of the social order of the family* may offer an understanding of concealment that goes beyond personality and disease (Goffman, 1959/1990, 1971/2010; Smart, 2007, 2011). Drawing attention to cultural norms for social encounters, particularly regarding pursuit and performance of normality, we are able to recognize the children's extensive contributions in families with alcohol problems and the consequences of this. Moreover, our analysis offers conceptual tools to understand the social interaction in these families as something else than co-dependency, which refers to psychopathological needs rather than the social aspects of the situation. The struggles of the children to perform normality in a challenging situation may be regarded as paradoxical potentials rather than pathology, demonstrating how social perspectives provide access to interpretations beyond the medical or psychological gaze.

### The family teamwork converting backstage havoc to front stage normality

Our analysis demonstrated how the problem-drinking parents’ manners of conduct caused disorder in the families, violating normative rules for actions as responsible parents and adult person. Dramaturgical metaphors offered insight in what was at stake and the specific efforts of the children to conduct the situational improprieties within and outside the family (Goffman, 1959/1990). Our interpretation is that the children's actions intended to re-establish social order within the family, to protect interactional disorder in public situations and to protect the public identity of their parents, themselves and the family as well. Additionally, the participants’ accounts showed parents who struggled to perform as ordinary parents and persons. The other parents’ apparently non-responsive conduct might also be intended to maintain social order, performing an agreement about a normal situation under control.

The stories demonstrate a heavy burden of responsibility for children, leading to a lot of needs, dilemmas and challenges. The efforts described in the participants’ accounts illustrated how the children belonged to a family team trying to comply with the conduct and expectations of the problem-drinking parents. To maintain or reconstruct social order they strived to do the remedial face-work when their parents refused to participate (Goffman, 1959/1990). The dramaturgical loyalty enacted by concealment in the front stage territories, kept the problems out of sight, but also impeded possibilities of the children's needs to be met. Furthermore, concealment offered the children the power to maintain dignity and prevent marginality. Similar normalization strategies have been described among children of parents with mental disorders (Fjone, Ytterhus, & Almvik, 2009; Trondsen, 2012; Trondsen & Tjora, 2014).

In public, the children performed according to an idealized version of the family and what is expected of a normally functioning family. We do not oppose a conclusion that such patterns may be regarded as dysfunctional. Yet, our findings suggest that such conceptual stereotypes are hardly helpful for individualized support. Service providers, aiming for recognition of families in need of support, should take the struggle for performance of normality that we have described as specific cues to recognize families where the social order might be disrupted, even when the public image appears normal.

### The family secret and the costs of disclosure

According to family sociologists, home is not only a place for love and domestic work, but also a potential space for oppression and violence (Smart, 2007). Recent contributions to family sociology have shifted attention from structure and function towards meaning and process (Wilson et al., 2012). Our study revealed how and why family secrets about problem drinking are maintained, aiming for performance as a normal family (Fjone et al., 2009; Trondsen, 2012; Trondsen & Tjora, 2014). Some of the participants in our study explained how disclosure would portray a negative image of an abnormal family, potentially offending their parents as well as themselves. Their accounts illustrated the everyday work of families and the ways in which family stories and secrets were managed, sticking to stories about a good family and neglecting to mention the negative, while facing an increasing gap between what they hoped for and their actual experiences(Goffman, 1971/2010; Smart, 2011). Performing an idealized impression of the family in public becomes a moral responsibility for the children (Goffman, 1959/1990; Lemert & Branaman, 1997). Thus, the family drama described by the participants became an unspeakable pain in childhood.

Sociology of childhood has drawn attention to how children are placed and perceived within social structures, while at the same time being agents and co-constructors of their social world (Brady et al., 2015). While it may not be an easy decision for a spouse to stay or leave their partners, children do not even have such a choice and are dependent on the parents in all respects. Our findings reveal children's uncertainty about whether the parents actually had an alcohol problem. Smart argues that individuals’ personal lives must be understood as related and embedded practices (Smart, 2007). However, there is still an urgent need to increase the awareness of the needs of all family members to be able to access appropriate help in their own right. An increased emphasis on the role of families and social networks in medical and social services is therefore necessary to support children in families with alcohol problems, aiming for prevention and reduction of harmful outcomes (Copello & Orford, 2002; Velleman, 2010).

### How can the family drama and the needs of children be recognized by professionals?

Our analysis indicates that children of parents with alcohol problems may suffer from unmet needs for professional support. It is a challenge for service providers to help families or individuals who work hard to appear as a normal family (Balsa et al., 2009; Burke et al., 2006). Several studies has focused on practice dilemmas faced by social welfare professionals working with families at risk (Forrester, Westlake, & Glynn, 2012; Taylor & Kroll, 2004). Taylor and Kroll (2004) emphasize the difficult balancing act required by professionals to develop a helping alliance with parents with alcohol and substance abuse problems, including denial, resistance, and a complex family dynamic (Taylor & Kroll, 2004). The secrecy surrounding the problems is not necessary in the children's interests, but indicates that they are trapped in a situation where it is impossible to ask for help (Kroll, 2004). When it comes to children of patients with alcohol problems, Norwegian law obliges professionals to have an awareness and, if necessary, intervene without request (Norwegian Directorate of Health, 2010). The findings we have presented emphasize that professionals must increase their awareness of families where parents may struggle and need help. Understanding that human beings typically want to present their best possible version of self and family, without thereby being labelled as co-dependent, may help professionals to sense alternative stories about families, beyond the immediate impression.

Supported by Goffman's theoretical perspectives about dramaturgy, backstage and frontstage, our analysis has elucidated the considerable efforts invested by the whole family to keep the family secret invisible for the surroundings and the different reasons for this. The interpretations and findings explain why it is so difficult to identify these families and their problems, but our data provide limited indications on how to facilitate discovery. However, the question may be further pursued in data from contexts where service providers actually accessed the scene. We will return to these questions in a later article.

Professionals need training in how to recognize children's concerns or adverse stories to be able to support them and their families. Furthermore, they need preparation in how to approach the practical and ethical dilemmas with parents who neither experience their alcohol use as a problem nor want help.

## Implications

Children of parents with alcohol problems work hard to perform normality aiming to restore social order and family interaction. Doing so, they attempt to protect themselves, their parents and the family identity, and the general public to prevent social breakdown. These normalization strategies also play a part to cover the children's unmet needs for professional support. Professionals aiming to identify and support this group of children should make every effort to recognize these strategies and understand their meanings to establish working alliances to support children and their families, rather than interpreting this pattern of behaviour as co-dependency.
